# Early Initiation of Sodium-Glucose Cotransporter 2 Inhibitors in Acute Decompensated Heart Failure: Efficacy, Safety, and Implementation in Stabilized Patients

**DOI:** 10.31083/RCM53604

**Published:** 2026-09-20

**Authors:** Alfredo Mauriello, Adriana Correra, Anna Chiara Maratea, Valeria Cetoretta, Francesco Giallauria, Giovanni Esposito, Alfonso Desiderio, Francesco Sabatella, Renato Gioia, Gemma Marrazzo, Biagio Liccardo, Vincenzo Russo, Paolo Trambaiolo, Antonello D’Andrea

**Affiliations:** ^1^S.C. Cardiology, Institute National Cancer, IRCCS, Fondazione “G. Pascale”, 80131 Naples, Italy; ^2^Cardiology Department, Ospedali Riuniti University Hospital, 71122 Foggia, Italy; ^3^Department of Cardiovascular Disease, ASL Napoli 1 Centro, 80145 Napoli, Italy; ^4^Cardiology and Arrhythmology Clinic, Marche Polytechnic University, University Hospital “Ospedali Riuniti”, 60126 Ancona, Italy; ^5^Department of Translational Medical Sciences, “Federico II” University of Naples, 80131 Naples, Italy; ^6^Cardiology and Intensive Care Unit, Department of Cardiology, “Umberto I” Hospital, 84014 Nocera Inferiore, Italy; ^7^Cardiology Unit, Department of Medical and Translational Sciences, University of Campania “Luigi Vanvitelli”, “V. Monaldi” Hospital, 80131 Naples, Italy; ^8^Intensive Cardiac Care Unit, Department of Cardiology, Sandro Pertini Hospital, 00157 Rome, Italy

**Keywords:** sodium-glucose cotransporter 2 inhibitors, intensive cardiac care, dapagliflozin, empagliflozin, sotagliflozin, heart failure

## Abstract

Sodium-glucose cotransporter 2 (SGLT2) inhibitors have fundamentally reshaped the pharmacological management of chronic heart failure (HF). However, the role of these inhibitors during the vulnerable phase of acute decompensated heart failure (ADHF) has historically been limited by concerns regarding hemodynamic stability and renal function. Recent clinical evidence suggests that the intensive care unit (ICU) and coronary care unit (CCU) may be optimal settings for initiating these disease-modifying therapies. This narrative review aims to evaluate the safety, efficacy, and clinical impact of early in-hospital initiation of SGLT2 inhibitors in patients admitted for ADHF, focusing on hemodynamic recovery, decongestion, and prevention of early readmission. Early initiation of SGLT2 inhibitors is associated with significant clinical benefits, including rapid decongestion, shorter length of hospital stay, and a reduction in the 30-day composite endpoint of death or worsening HF. Furthermore, SGLT2 inhibitors exhibit synergistic effects with loop diuretics, helping to overcome diuretic resistance. Safety profiles remain favorable when patients satisfy basic clinical stability criteria. The traditional wait-and-see approach to initiating SGLT2 inhibitors is being replaced by a proactive early-start strategy. In the cardiac intensive care setting, SGLT2 inhibitors are no longer viewed solely as metabolic agents but also as tools for acute hemodynamic optimization. Further research is required to refine the management of euglycemic ketoacidosis and to determine the optimal timing of initiation in patients requiring mechanical circulatory support.

## 1. Introduction

Heart failure (HF) is a clinical syndrome caused by a structural and/or functional cardiac abnormality and resulting in reduced cardiac output (CO) and/or elevated intracardiac pressures at rest or during stress, which could determine the development of typical symptoms, such as breathlessness, ankle swelling, and fatigue, that may be associated with signs, such as elevated jugular venous pressure, pulmonary crackles, and peripheral oedema [1,2]. The incidence of HF is approximately 3–5 per 1000 person-years. The HF prevalence is approximately 1–2% in adults, and it increases with age, accounting for up to 10% in patients aged 70 years or over [1]. Acute heart failure (AHF) is broadly defined as a rapid onset of new or worsening signs and symptoms of HF [3]. De novo AHF results from a sudden increase in intracardiac filling pressures and/or acute myocardial dysfunction, which can lead to decreased peripheral perfusion and pulmonary oedema [4]. The most common aetiology is cardiac ischaemia [3]. A less common precipitant of AHF is one of the possible non-ischaemic myocardial insults [3]. Most patients presenting with AHF do so in the context of pre-existing cardiomyopathy, a situation described as acute decompensated heart failure (ADHF). Approximately 25–40% of AHF cases present as de novo episodes, while the remaining 60–75% consist of acute decompensated chronic heart failure (CHF) [5]. In-hospital mortality ranges from 4% to 7%, with a median length of stay between 4 and 11 days [5]. Post-discharge mortality continues to be elevated, in the range of 20–36% [6,7].

Sodium-glucose cotransporter 2 inhibitors (SGLT2i) are selective inhibitors of sodium-glucose cotransporter 2 (SGLT2) that block glucose reabsorption in the proximal tubule of the kidney and promote glucosuria [8,9,10,11]. SGLT2i were originally developed for the treatment of type 2 diabetes mellitus (T2DM), and pleiotropic effects on the cardiovascular and renal systems were soon highlighted through inflammation, oxidative stress, cellular energy metabolism, and housekeeping mechanisms [12,13]. They represent one of the four pillars for CHF [1,14]. Our comprehensive review aims to evaluate the safety, efficacy, and clinical impact of early in-hospital initiation of SGLT2i in patients admitted for AHF and ADHF, with a focus on hemodynamic recovery, decongestion, and prevention of early readmission.

## 2. Materials and Methods

This narrative review provides a comprehensive synthesis and critical analysis of current knowledge, clinical advancements, and evolving strategies concerning the in-hospital initiation of SGLT2i in patients admitted for ADHF. Narrative reviews are particularly effective for synthesizing broad research domains that undergo rapid paradigm shifts, such as the current transition from a wait-and-see approach to a proactive early-start strategy in the cardiac intensive care setting. To identify relevant sources, a structured literature search was conducted focusing on the safety, efficacy, and hemodynamic impact of SGLT2i during the vulnerable phase of HF.

The search strategy used biomedical databases, including PubMed/MEDLINE and EMBASE, to retrieve publications from January 2015 to March 2026, adhering to standard practices for evidence-based reviews in cardiovascular medicine. The search strings and key terms employed for literature collection included combinations of the following concepts, utilizing Boolean operators (AND/OR):

Primary Condition: “Acute decompensated heart failure” (ADHF), “Acute heart failure” (AHF), “Congestion”. Clinical Setting: “Intensive Care Unit” (ICU), “Coronary Care Unit” (CCU), “Cardiac Intensive Care Units” (CICUs), “In-hospital initiation”.

Therapeutic Strategies: “SGLT2 inhibitors” (SGLT2i), “Empagliflozin”, “Dapagliflozin”, “Sotagliflozin”, “Sequential nephron blockade”, “Diuretic synergy”.

Document selection prioritized English-language articles, including randomized controlled trials (RCTs), which serve as the three pillars of current clinical evidence. The review also encompasses retrospective studies and expert consensus focused on the management of specific complications, such as euglycemic ketoacidosis and the use of SGLT2i in patients requiring mechanical circulatory support (MCS). This methodology ensures that the narrative synthesis is evidence-based and reflects the transition of SGLT2i from metabolic drugs to essential tools for acute hemodynamic optimization.

## 3. Hemodynamic Effects of SGLT2i

SGLT2i do not function merely as metabolic drugs; they exert rapid and significant hemodynamic effects useful in the management of AHF. The primary pathophysiological mechanisms of action in the acute setting are detailed below:

### 3.1 Direct Myocardial Effects of the SGLT2i

Alongside the systemic hemodynamic effects mediated by renal action, emerging evidence indicates that SGLT2i also exert significant direct protective effects on cardiac cells, despite the myocardium being largely devoid of the SGLT2 transporter under normal conditions [15]. This mechanism of action focuses on the regulation of ionic homeostasis through the so-called cardiac sodium-interactome [15]. Under the influence of pathological stimuli characteristic of HF, such as hypoxia, acidosis, and inflammation, SGLT2i directly inhibit the sodium-hydrogen exchanger (NHE-1) and the late sodium current (late- $I_{\text{Na}}$ ) on the cell membrane. The resulting reduction in intracellular sodium levels ( ${\left[Na^{+}\right]}_{i}$ ) subsequently prevents cytosolic calcium overload, reduces mitochondrial oxidative stress, and attenuates NLR Family Pyrin Domain Containing 3 (NLRP3) inflammasome activation, directly contributing to the improvement of myocardial contractile and diastolic function independently of systemic diuretic action [15]. The existence of these direct myocardial pathways, which are completely independent of estimated glomerular filtration rate (eGFR), provides a solid biological rationale for the use of SGLT2i even in patients undergoing chronic dialysis, in whom molecular cardiovascular benefits persist despite the loss of renal organ function [16].

### 3.2 Diuretic and Natriuretic Effects: Sequential Nephron Blockade

SGLT2i act primarily in the proximal tubule, where the bulk of sodium reabsorption occurs [17].

When added to loop diuretics, SGLT2i enhance sodium and water excretion through sequential nephron blockade [18]. This mechanism is particularly effective in overcoming diuretic resistance. While the co-administration of thiazide and loop diuretics facilitates superior natriuresis in cases of refractory congestion, this sequential nephron blockade often precipitates a decline ineGFR, profound electrolyte imbalances, and detrimental neurohormonal up-regulation [19]. Conversely, SGLT2i offer a more favorable diuretic profile. By promoting electrolyte-free water clearance, the integration of SGLT2i into intravenous loop diuretic regimens achieves potent osmotic diuresis while preserving ionic stability and attenuating the compensatory activation of the renin-angiotensin-aldosterone and sympathetic nervous systems [20,21]. SGLT2i increase 24-hour urine volume by approximately 500 mL or an 20–30% of increasing [17].

Emerging evidence from pilot studies indicates that SGLT2i possess diuretic properties that complement, rather than merely replicate, those of conventional loop diuretics. A distinguishing feature of their mechanism is a targeted reduction in interstitial fluid, which appears to spare the intravascular volume. By prioritizing tissue decongestion over systemic depletion, this class of drugs may optimize tissue perfusion and stabilize renal hemodynamics, offering a more nuanced approach to volume management [22,23,24].

Finally, in patients with HF and loop diuretic resistance, dapagliflozin was no more effective than metolazone in relieving congestion. Patients assigned to dapagliflozin received a larger cumulative dose of furosemide but experienced less biochemical upset than those assigned to metolazone, such as hyponatremia or hypokalemia and a lower incidence of significant rises in urea and creatinine levels [23].

### 3.3 Reduction of Filling Pressures: Preload and Afterload Optimization

SGLT2i facilitate a rapid optimization of cardiac loading conditions through several distinct pathways. Induced osmotic diuresis effectively reduces plasma volume, thereby decreasing preload, while a modest reduction in systemic blood pressure contributes to a lowered afterload [25]. A clinical advantage is that these hemodynamic shifts occur without triggering the sympathetic nervous system or the renin-angiotensin-aldosterone system (RAAS), a common complication observed with thiazide diuretics [20,21,26]. Corroborating these hemodynamic effects, Bogoviku et al. [25] in a sub-analysis of the Early Empagliflozin Initiation on Diuresis and Kidney Function in Patients With Acute Decompensated Heart Failure (EMPAG-HF) trial, including 60 patients with AHF (mean age 74.7 ± 9.9 years; 62% male), showed that after five days of treatment, the empagliflozin cohort exhibited a significant reduction in both left atrial volume (LAV) (*p* < 0.001) and left atrial end-systolic volume index (LAESVI) (*p* = 0.016) compared to the placebo group.

### 3.4 Renal Protection and Tubulo-Glomerular Feedback

The initial renal response to SGLT2i is frequently characterized by a modest decline in the eGFR, commonly referred to as the “eGFR dip”, this early decline is a functional hemodynamic shift rather than structural injury [27].

By inhibiting sodium reabsorption in the proximal tubule, SGLT2i increase sodium delivery to the macula densa [27]. This triggers the tubuloglomerular feedback mechanism, leading to afferent arteriolar vasoconstriction [27]. This vasoconstriction alleviates hyperfiltration and reduces pressure within the glomerulus, which manifests clinically as the initial eGFR dip [27]. It serves as a precursor to superior long-term renal preservation and a reduction in albuminuria. Increases in serum creatinine of up to 25% from baseline are considered clinically acceptable and do not warrant drug discontinuation [28]. The early initiation of SGLT2i during hospitalization for AHF is safe [29]. It promotes effective decongestion while simultaneously shielding the kidney from barotrauma and facilitating the introduction of other life-saving therapies.

## 4. Practical Management of SGLT2i in Acute Setting

The clinical implementation of SGLT2i in early post-decompensated patients necessitates a balanced approach: identifying the optimal window for stabilization while maintaining rigorous monitoring to capitalize on early benefits without compromising safety.

### 4.1 Stability Criteria for Initiation

Initiating SGLT2i during AHF hospitalization is safe provided the patient has achieved hemodynamic stability. Current clinical protocols typically require:

A stable systolic blood pressure (SBP), with thresholds usually set at >90 mmHg or >110 mmHg in more conservative settings [30].

Patients must be off intravenous inotropes. Those in cardiogenic shock or exhibiting signs of active hypoperfusion are not immediate candidates [30].

Therapy can commence once pulmonary congestion is alleviated, even as the patient transitions from intravenous to oral diuretic regimens [30].

A eGFR >25–30 mL/min/1.73 m^2^ and stable potassium levels are required, given the natriuretic synergy with loop diuretics [30].

### 4.2 Monitoring: The eGFR Dip and Euglycemic Ketoacidosis

Safety monitoring should focus on two primary clinical markers: renal function and metabolic stability.

ADHF is frequently characterized by systemic acidosis, primarily lactic acidosis due to peripheral tissue hypoperfusion, or metabolic acidosis secondary to severe renal congestion and acute kidney injury (AKI). In this fragile setting, the known class-related risk of euglycemic diabetic ketoacidosis (EDKA) associated with SGLT2i, combined with the expected initial eGFR dip, requires careful clinical contextualization. Analysis of the renal and metabolic profiles across Dapagliflozin In Acute Heart Failure (DICTATE-AHF), A Study to Test the Effect of Empagliflozin in Patients Hospitalized for Acute Heart Failure (EMPULSE) and Effect of Sotagliflozin on Cardiovascular Events in Patients With Type 2 Diabetes Post Worsening Heart Failure (SOLOIST-WHF) demonstrates that the intersection between heart failure-induced acidosis and SGLT2i initiation does not translate into a clinical hazard (Table 1 Ref. [31,32,33]).

**Table 1.** **Baseline renal function and eGFR “dip” dynamics across the highlighted trials**.

	Baseline eGFR (mL/min/1.73 m^2^)	Immediate eGFR “dip”/drop	Baseline acidosis	Long-term renal clinical outcome
SOLOIST-WHF [31]	Median: 49.0 (IQR: 36.0–66.0)	Initial transient eGFR dip observed post-initiation	Not present/not reported. Patients were stabilized post-worsening HF prior to or shortly after discharge, meaning severe systemic acidosis was not a feature at baseline.	Did not translate into long-term renal injury; demonstrated sustained preservation of renal function over time
EMPULSE [32]	Mean: 53.8 ± 20.3	Early functional eGFR dip occurred within the first days	Not present/not reported. Enrolled strictly hemodynamically stabilized patients, where acute metabolic/lactic acidosis was resolved or absent.	Resolved by day 30. Treatment led to less frequent acute renal failure events vs. placebo (7.7% vs. 12.1%)
DICTATE-AHF [33]	Mean: 56.3 ± 22.3	Transient functional decline observed during acute IV diuresis	Not present/not reported. Within 24 hours of hospital presentation, excluding those with acute tissue hypoperfusion or shock.	No significant difference in worsening renal function or incidence of AKI compared to usual care.

DICTATE-AHF, Dapagliflozin In Acute Heart Failure; eGFR, estimated glomerular filtration rate; EMPULSE, A Study to Test the Effect of Empagliflozin in Patients Hospitalized for Acute Heart Failure; HF, heart failure; IQR, interquartile range; SOLOIST-WHF, Effect of Sotagliflozin on Cardiovascular Events in Patients With Type 2 Diabetes Post Worsening Heart Failure; AKI, acute kidney injury; IV, intravenous.

It is common to observe an initial decline in eGFR of approximately 2–4 mL/min/1.73 m^2^ during the first weeks [34]. This dip is a functional result of tubuloglomerular feedback reducing intraglomerular pressure, not a sign of acute kidney injury (AKI) [34]. This initial drop tends to stabilize and disappear within 90 days [34]. A decline exceeding 50%, however, mandates immediate clinical reassessment. However, empagliflozin significantly reduced parameters of AKI (urinary tissue inhibitor of metalloproteinases 2 (TIMP-2) and insulin-like growth factor-binding protein 7 (IGFBP7) by NephroCheck® as indicators of tubular kidney damage), which became significant after 3 days of treatment (*p* = 0.02) and remained significant at the 7-day time point (*p* = 0.003), suggesting a nephroprotective effect despite the decline in eGFR [34].

Though rare, EDKA must be suspected in patients presenting with nausea, vomiting, or malaise, even if blood glucose levels appear normal. High-risk scenarios include T2DM patients under extreme physiological stress or prolonged fasting [33]. If suspected, SGLT2i must be suspended, and blood pH, ketones, and bicarbonate levels must be evaluated [33]. In ICU, consider a sick day protocol involving the temporary suspension of SGLT2i during periods of prolonged fasting, major surgery, or severe sepsis to mitigate EDKA risk.

### 4.3 Synergistic Effects With Loop Diuretics

The co-administration of SGLT2i and loop diuretics creates an adaptive natriuretic synergy [24]. Early SGLT2i introduction often allows for lower daily doses of loop diuretics by enhancing the natriuretic response without worsening renal function [24].

While severe dehydration is rare, close monitoring of volume status is essential [24]. Furthermore, the combined therapy may increase potassium excretion; therefore, regular serum potassium checks are recommended during the co-administration phase [24,31]. Table 2 represents the main parameters for management of early post-decompensated patients, during SGLT2i administration. Fig. 1 represents the SGLT2i monitoring protocol during the first 72 hours of admission. Table 3 represents the ICU monitoring checklist during the first 72 hours.

**Table 2.** **Main parameters to evaluate during administration of SGLT2i during AHF**.

Parameter	Threshold/target	Clinical pearl
Timing	24–96 hours post-stabilization	Reduces hospital stay and improves post-discharge outcomes
SBP	>90 mmHg	Ensure stability without vasopressors
Creatinine	Up to 25% rise	Considered functional and acceptable
Potassium	Baseline stability	Monitor closely when combined with loop diuretics

AHF, acute heart failure; SBP, systolic blood pressure; SGLT2i, sodium-glucose cotransporter 2 inhibitors.

**Table 3.** **ICU monitoring checklist during first 72 hours**.

Parameter	Frequency	Target/action
Creatinine	Daily	An increase of up to 25% from baseline may be acceptable and does not necessarily warrant discontinuation of SGLT2i; continue clinical monitoring.
eGFR	Daily	An initial functional decline may occur after SGLT2i initiation; reassess the patient if eGFR decreases by >50% from baseline.
Serum potassium	Every 12–24 h	Monitor for hypokalemia when combined with high-dose loop diuretics
Blood glucose	Every 8–12 h	Monitor for euglycemic ketoacidosis even if glucose is normal
Urine output	Continuous	Expect a 20–30% increase in volume or 500 mL; adjust loop diuretic dose if needed
Euglycemic ketoacidosis	Every 8–12 h	If nausea, vomiting, or malaise occur: stop SGLT2i immediately and measure pH, ketones, and bicarbonate
Blood pressure	Continuous	Ensure SBP remains >90 mmHg

ICU, Intensive Care Unit; SGLT2i, Sodium-Glucose Cotransporter 2 inhibitors.

**Fig. 1.**
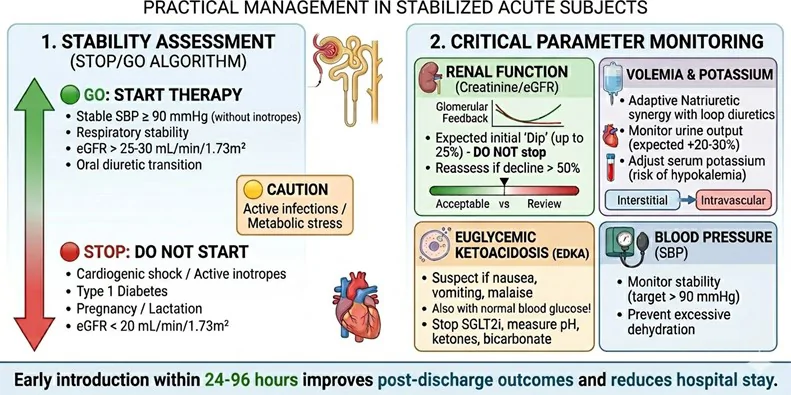
**SGLT2i monitoring protocol during the first 72 hours of admission**. eGFR, estimated glomerular filtration rate; SGLT2i, sodium-glucose cotransporter 2 inhibitors; SBP, systolic blood pressure.

## 5. Current Evidence Regarding Use of SGLT2i during AHF

Use of SGLT2i during AHF is established by several trials summarized in Table 4.

**Table 4.** **Comparative analysis of major SGLT2i trials in AHF**.

Feature	DICTATE-AHF	EMPULSE	SOLOIST-WHF
Drug	Dapagliflozin (10 mg)	Empagliflozin (10 mg)	Sotagliflozin (200–400 mg)
Study population	240 patients with AHF and hypervolemia	530 patients hospitalized for AHF (de novo or chronic)	1222 patients with T2DM recently hospitalized for HF
Timing of initiation	Ultra-early: within 24 h of hospital presentation	In-hospital: median 3 days after stabilization	Pre/Post-discharge: median 2 days post-discharge.
Primary focus	Diuretic efficiency: natriuresis and weight loss	Clinical benefit: Win Ratio (death, HF events, KCCQ)	CV death and urgent HF visits
Diabetic status	Diabetic and non-diabetic	Diabetic and non-diabetic	T2DM only
Ejection fraction	Primarily focused on congestion	HFrEF, HFmrEF, HFpEF	All EF with consistent benefit in HFpEF
Key results	Significant diuretic sparing effect; increased 24 h natriuresis	36% higher likelihood of clinical benefit at 90 days	33% reduction in the primary composite endpoint
Safety		No adverse effect on electrolyte balance compared to placebo	No adverse effect on electrolyte balance compared to placebo
Clinical takeaway	Enhances early decongestion and reduces loop Diuretic need	Safely improves symptoms and congestion markers early on	Reduces the high risk of re-hospitalization in the vulnerable phase

AHF, acute heart failure; CV, cardiovascular; HF, heart failure; HFmrEF, heart failure with mildly reduced ejection fraction; HFpEF, heart failure with preserved ejection fraction; HFrEF, heart failure with reduced ejection fraction; T2DM, type 2 diabetes mellitus; KCCQ, Kansas City Cardiomyopathy Questionnaire; SOLOIST-WHF, Effect of Sotagliflozin on Cardiovascular Events in Patients With Type 2 Diabetes Post Worsening Heart Failure; EF, ejection fraction.

DICTATE-AHF Trial [33] evaluates the efficacy and safety of early dapagliflozin initiation in patients with AHF compared to standard care. It is a multicenter, open-label study, including 240 patients (mean age 64.5, with range of 55–74 years; 60.9% male), that were randomized within 24 hours of hospital presentation for hypervolemic AHF to dapagliflozin 10 mg once daily or structured usual care with protocolized diuretic titration until day 5 or hospital discharge. Dapagliflozin was associated with reduced loop diuretic doses (*p* = 0.006) and fewer intravenous diuretic up-titrations (*p* ≤ 0.05) to achieve equivalent weight loss as usual care, although it did not meet its primary endpoint of diuretic efficiency (diuretic efficiency calculated as cumulative weight change divided by cumulative loop diuretic dose and expressed as kg/40 mg IV furosemide-equivalent, for dapagliflozin vs. placebo at 5 days, *p* = 0.06). In addition, Dapagliflozin was safe, and it was associated with improved median 24-hour natriuresis (*p* = 0.03) and urine output (*p* = 0.005), expediting hospital discharge over the study period.

SOLOIST-WHF Trial [31] showed that initiation of Sotagliflozin in AHF in stabilized patients prior to discharge or shortly thereafter is safe and effective. The benefits were consistent in those with HF with reduced but also preserved ejection fraction. It is a multicenter, double-blind trial in which 1222 patients (median age was 70 years, 66.3% were male) exclusively with T2DM who were recently hospitalized for worsening HF were randomly assigned to receive Sotagliflozin or placebo. The authors demonstrated that the primary end point, the total number of deaths from cardiovascular causes and hospitalizations and urgent visits for HF (first and subsequent events), was lower in the Sotagliflozin group compared to controls (*p *< 0.001).

EMPULSE Trial [32], including 530 patients hospitalized for AHF, strictly patients who were already hemodynamically stabilized (mean age 71, with range 61–78 years; 66.2% male). The outcomes investigated were: weight loss (WL), WL adjusted for mean daily loop diuretic dose (WL-adjusted), area under the curve of change from baseline in N-terminal pro-B-type natriuretic peptide levels, hemoconcentration, and clinical congestion score, Kansas City Cardiomyopathy Questionnaire (KCCQ) Total Symptom Score after 15, 30, and 90 days of treatment. Compared with placebo, patients treated with empagliflozin demonstrated significantly greater reductions in all studied markers of decongestion at all time points (all *p* < 0.05).

As detailed in Table 5 (Ref. [31,32,33]), the vast majority of patients enrolled across these landmark trials were already receiving an optimized, comprehensive medical regimen for HF. Notably, the clinical benefits of SGLT2i on cardiovascular mortality and HF hospitalizations were demonstrated on top of these foundational baseline therapies, including high rates of RAAS inhibitors, beta-blockers, and mineralocorticoid receptor antagonists (MRAs). This underscores that SGLT2i do not merely act as alternative diuretic agents, but rather function synergistically within the totality of the modern quadruple-therapy regimen, even in the highly vulnerable phases of acute and recently worsening heart failure.

**Table 5.** **Background heart failure therapy across the highlighted landmark trials**.

Study	Diuretics	RAAS inhibitors (ACEi/ARB/ARNI) (%)	Beta-blockers (%)	MRAs (%)
SOLOIST-WHF [31]	99.1%	85.9% (ACEi/ARB: 68.3% / ARNI: 17.6%)	88.9%	49.3%
EMPULSE [32]	90.1%	78.4% (ACEi/ARB: 60.1% / ARNI: 18.3%)	81.3%	48.0%
DICTATE-AHF [33]	100%	70.6% (ACEi/ARB: 44.4% / ARNI: 19%)	85%	41%

ACEi, Angiotensin-Converting Enzyme Inhibitors; ARB, Angiotensin II Receptor Blockers; ARNI, Angiotensin Receptor-Neprilysin Inhibitors; DICTATE-AHF, Dapagliflozin In Acute Heart Failure; EMPULSE, A Study to Test the Effect of Empagliflozin in Patients Hospitalized for Acute Heart Failure; MRA, Mineralocorticoid Receptor Antagonists; RAAS, Renin-Angiotensin-Aldosterone System; SOLOIST-WHF, Effect of Sotagliflozin on Cardiovascular Events in Patients With Type 2 Diabetes Post Worsening Heart Failure.

Regarding the optimal timing and sequencing of SGLT2i within the modern foundational ‘four-pillar’ regimen, the actual clinical paradigm shifts away from the traditional, slow step-wise titration. Instead, we propose a simultaneous or rapid-sequence initiation protocol.

Rather than delaying SGLT2i therapy until afterload-reducing agents or beta-blockers are fully uptitrated, SGLT2i should be introduced at the same time or within the first 24–48 hours of stabilizing foundational therapy [29]. The rationale for this early placement is twofold: first, SGLT2i do not significantly impact blood pressure or heart rate, making them exceptionally well-tolerated even when other agents are being introduced; second, their osmotic and natriuretic effects can facilitate the optimization of loop diuretics, preventing the reflex neurohormonal activation often triggered by high-dose diuretic monotherapy.

Taken together, these studies support a consistent message: early initiation of SGLT2ifollowing an episode of AHF appears safe and may confer clinically relevant benefits, particularly in terms of decongestion and reduction in HF-related events. However, the magnitude and nature of benefit vary across studies, with DICTATE-AHF showing mainly physiological improvements, and SOLOIST-WHF and EMPULSE demonstrating reductions in clinical events, largely driven by HF hospitalizations rather than mortality.

Despite these encouraging findings, a critical limitation must be emphasized. None of these trials enrolled patients in the truly acute, unstable phase of HF, such as those with cardiogenic shock, ongoing hypoperfusion, or requiring inotropic or vasopressor support. Instead, all three studies systematically selected hemodynamically stabilized patients, based on protocol-defined inclusion criteria. As a consequence, the available evidence does not provide validated clinical or laboratory thresholds to guide the initiation of SGLT2i in critically ill or ICU populations. Reported criteria—such as systolic blood pressure thresholds, absence of intravenous therapies, or minimum eGFR levels—should be interpreted as trial eligibility requirements rather than evidence-based treatment algorithms.

Therefore, while current data support the safety of early SGLT2i initiation in stabilized AHF patients, the optimal timing of therapy in the acute and unstable setting remains undefined. In the absence of dedicated studies in critically ill populations, it is only possible to derive pragmatic, hypothesis-generating approaches based on trial inclusion criteria. These may inform the development of provisional flowcharts for clinical practice, emphasizing the need for prior hemodynamic stabilization, absence of ongoing intravenous support, and adequate renal function before treatment initiation.

## 6. Future Perspectives

Future studies specifically targeting ICU populations and incorporating detailed hemodynamic and biochemical profiling will be essential to define evidence-based thresholds and timing strategies for SGLT2i initiation in AHF.

The use of SGLT2i in patients supported by Impella or Extracorporeal Membrane Oxygenation (ECMO) remains an evolving frontier. While they may facilitate weaning by optimizing preload, the risk of EDKA in hypermetabolic, critically ill patients necessitates cautious monitoring. The wait-and-see approach persists here, pending further safety data regarding hemodynamic stability during MCS. In patients supported by MCS, standard blood glucose monitoring is insufficient. We suggest implementing a routine ketone monitoring protocol in blood or urine to ensure safety, as EDKA can occur even with near-normal glycemic levels.

In addition, SGLT2i have bridged a significant therapeutic gap in heart failure with preserved ejection fraction (HFpEF). Evidence from the Empagliflozin Outcome Trial in Patients with Chronic Heart Failure with Preserved Ejection Fraction (EMPEROR-Preserved) and Dapagliflozin Evaluation to Improve the Lives of Patients with Preserved Ejection Fraction Heart Failure (DELIVER) trials confirms that their clinical benefit extends across the entire left ventricular ejection fraction spectrum. In the acute setting, early initiation in HFpEF patients is important to prevent early hospitalizations, leveraging their unique ability to improve diastolic stiffness and systemic congestion.

A pivotal challenge in the acute setting remains the accurate differentiation between the functional eGFR dip and true structural AKI. Future management strategies in the ICU will likely integrate cell-cycle arrest biomarkers, such as TIMP-2 and IGFBP7, into routine clinical practice. These markers can provide real-time insights into tubular integrity, allowing clinicians to distinguish benign hemodynamic shifts from cellular damage. This precision medicine approach will be essential to prevent the unnecessary discontinuation of SGLT2i, ensuring that patients maintain the long-term nephroprotective benefits of these agents even after an initial decline in renal function.

In addition, the optimal chronological sequencing and timing relative to the other three pillars remains undefined. Future research must clarify whether simultaneous quadruple-pillar initiation within the first 48 hours of stabilization is superior to rapid-sequence titration in terms of 30-day readmission rates, patient tolerance, and systemic neurohormonal mitigation.

## 7. Conclusions

The pharmacological management of AHF is undergoing a profound transformation, moving away from a conservative wait-and-see approach toward the proactive, early in-hospital initiation of SGLT2i. The introduction of SGLT2i within the first 24–96 hours of stabilization is not only safe but may provide clinical benefits, including accelerated decongestion, shorter hospital stays, and a robust reduction in the 30-day risk of death or worsening HF. Despite these advancements, further research is warranted to optimize the timing of initiation in patients requiring MCS and to refine safety protocols for the prevention of EDKA in critically ill populations.
